# Complete genome sequence of *Xanthomonas euvesicatoria* pv. *polysciadis* pathotype strain Pgu1^PT^, a phytopathogenic bacterium isolated from *Polyscias guilfoylei* (geranium aralia)

**DOI:** 10.1128/mra.00240-25

**Published:** 2025-06-17

**Authors:** Chien-Fu Wu, Shin Lee, Yi-Chiao Huang, Chia-Ching Chu

**Affiliations:** 1 Department of Plant Pathology, The Ohio State University 242626 , Wooster, Ohio, USA; 2 Department of Plant Pathology, National Chung Hsing University 34916, Taichung, Taiwan; University of Strathclyde, Glasgow, United Kingdom

**Keywords:** phytopathology, genome, *Xanthomonas*

## Abstract

Here, we report the complete genome sequence of *Xanthomonas euvesicatoria* pv. *polysciadis* strain Pgu1^PT^, a phytopathogenic bacterium causing necrotic leaf lesions on *Polyscias guilfoylei* in Taiwan. The genome consists of a 5,129,423 bp circular chromosome (GC content: 64.8%) and a 53,818 bp circular plasmid (GC content: 62.1%).

## ANNOUNCEMENT

*Xanthomonas euvesicatoria* is a phytopathogenic bacterial species including many host-specific pathovars (1). *Xanthomonas euvesicatoria* pv. *polysciadis* was recently found causing leaf blight of *Polyscias guilfoylei*, representing one of the first cases of *X. euvesicatoria* infecting Araliaceae plants (2). Since genomic data for this pathovar remain unavailable, we performed whole-genome sequencing on the pathotype strain Pgu1^PT^ using the Pacific Biosciences (PacBio) Sequel platform.

Pgu1^PT^ was obtained from a leaf lesion on *P. guilfoylei* collected in Taichung, Taiwan (24°08′50.5″N 120°38′01.4″E) in 2020 (2). The strain was isolated by streaking a suspension of minced lesion onto nutrient agar (NA; beef extract 0.3%, peptone 0.5%, and agar 1.5%, pH 7.0) and incubating the plates aerobically at 25°C for 72 h (2), followed by colony picking. The strain was preserved in 20% glycerol at −80°C.

For DNA extraction, Pgu1^PT^ was revived on NA (25°C), and 2-day-old cells were transferred to nutrient broth (5 mL) including 0.5% yeast extract and cultured for 1 day. Then, 50 µL of the culture was inoculated to 50 mL of the same medium and grown overnight at 25°C. The culture (50 mL) was centrifuged (10,000 × *g*, 5 min, and 4°C). Cells were resuspended in 5 mL of buffer (10 mM Tris-HCl, 0.1 mM EDTA, and 100 mM NaCl, pH 8.0), and DNA was extracted using phenol-chloroform as previously described (3). Approximately 1 µg of the DNA was fragmented using g-TUBE (Covaris), then purified with 0.45× AMPure PB beads (PacBio), retaining fragments above 1 kb. A SMRTbell library was prepared using the SMRTbell Express Template Prep Kit 2.0 (PacBio). Sequencing was conducted with PacBio Sequel (SMRT Cell 1M v3 Tray; v3 chemistry) by Genomics BioSci & Tech (Taiwan). Raw read quality control and adapter trimming were performed using SMRT Link version 9.0 (PacBio). The sequencing generated 450,613 subreads (*N*_50_: 4,059 bp), and processing with CCS version 6.0.0 (PacBio) resulted in 20,167 HiFi reads (*N*_50_: 3,979 bp). The reads were assembled using hifiasm version 0.15.3 (4) and polished using GCpp version 2.0.2 (PacBio) and Canu version 2.1 (5). Circlator version 1.5.5 was used to circularize the genome, setting the *dnaA* gene as the starting point (6). Circularity of plasmid was confirmed manually by identifying a 5,542 bp overlapping sequence at both the 5′ and 3′ ends in Geneious R10 (Biomatters Ltd.). The genome assembly was evaluated using QUAST version 5.0.2 (7), and completeness was assessed using BUSCO version 5.8.2 (xanthomonadales_odb12.2024-11-14 database) (8). Annotation was completed using NCBI’s Prokaryotic Genome Annotation Pipeline version 6.9 (9). Default parameters were used for all data analyses.

The final assembly (genome coverage: 12.7×) resulted in a single circular chromosome (single contig) with a size of 5,129,423 bp (GC content: 64.8%) and a 53,818 bp circular plasmid (GC content: 62.1%). BUSCO analysis revealed 99.5% completeness, 12 duplicated genes (1.3%), 1 missing gene (0.1%), and 4 fragmented genes (0.4%). Annotation resulted in 4,171 protein-coding genes, 53 tRNAs, 6 rRNAs, and 35 ncRNAs. The chromosomal sequence was analyzed on the Type Strain Genome Server version v391 (10). The closest strain was *X. euvesicatoria* pv. *perforans* CFBP 7293 (accession no. GCA_001976075; 88.6% dDDH-d4 value).

## Data Availability

The genome sequences of strain Pgu1^PT^ have been deposited in GenBank under the accession numbers CP180391 (chromosome) and CP180392 (plasmid). The raw reads have been deposited under SRA number SRR32212838, and the associated BioProject and BioSample numbers are PRJNA1217515 and SAMN46493364, respectively.
